# Population genetic characterization and family reconstruction in brood bank collections of the Indian major carp *Labeo rohita* (Cyprinidae:Cypriniformes)

**DOI:** 10.1186/s40064-015-1571-9

**Published:** 2015-12-14

**Authors:** Ashraf Ullah, Abhisak Basak, Md. Nazrul Islam, Md. Samsul Alam

**Affiliations:** Department of Fisheries Biology and Genetics, Bangladesh Agricultural University, Mymensingh, 2202 Bangladesh

**Keywords:** Microsatellite, Genetic variation, Major carps, Brood bank, Bottleneck, Sib-ship

## Abstract

The founder stock of a captive breeding program is prone to changes in genetic structure due to inbreeding and genetic drift. Genetic characterization of the founder population using suitable molecular markers may help monitor periodic changes in the genetic structure in future. To develop benchmark information about the genetic structure we analyzed six microsatellite loci in the Brodbank collections of rohu (*Labeo rohita*) originated from three major rivers—the Jamuna, the Padma and the Halda. A total of 28 alleles were detected in 90 individuals with an average of 4.6 alleles per locus. The average observed heterozygosity ranged from 0.655 to 0.705 and the expected heterozygosity ranged from 0.702 to 0.725. The mean *F*_*IS*_ values were 0.103, 0.106 and 0.018 for the Jamuna, Padma and Halda fishes respectively. The population pair-wise *F*_*ST*_ values ranged from 0.0057 to 0.0278. Structure analysis grouped the fishes of the three rivers into two clusters. The numbers of half-sib families were 5, 5 and 4 and the numbers of full-sib families were 12, 10 and 18 for the Halda, Jamuna and the Padma samples respectively. Bottleneck was detected in all the river samples. We recommend to collect more fish from different locations of the major rivers to broaden the genetic variability of the founder stocks of the Brood bank.

## Background

*Labeo rohita* (Hamilton), a member of the Indian major carps group of the family Cyprinidae is naturally distributed in the rivers of Bangladesh, India, Pakistan and Myanmar (Talwar and Jhingran 1991). The column feeder rohu (*L. rohita*), along with the surface feeder catla, *Catla catla* (Hamilton) and bottom feeder mrigal, *Cirrhinus cirrhosus* (Hamilton) constitutes an important and popular aquaculture practice in the region named carp polyculture system. *L. rohita* is the highest contributing single species in aquaculture production of Bangladesh sharing 22.0 % of the country’s pond production (FRSS 2012). The world aquaculture production of *L. rohita* was 1.17 million MT in 2010 approximately 2 % of the total aquaculture production that ranked 11th in the list of principal contributing species (FAO 2012).

Aquaculture in Bangladesh has experienced a steady growth of about 6 % since 1984/85 until present due to horizontal expansion as well as intensification and contributing more than 50 % to the total fish production of the country (FRSS 2012). Cheap and simple spawn production technology and attractive margin led to establishment of some 900 carp hatcheries in the country over the decades. However, hatcheries engaged in production of carp spawn are reported not to be managed properly due to multiple factors such as lack of awareness about the negative consequences of improper management, lack of technical knowledge, lack of facilities for maintaining required number of quality breeders and overall high competition in the fish seed market. Use of smaller size breeders (negative selection), inbreeding and hybridization have been common practices in the hatcheries that are individually or collectively responsible for the deterioration of fish seed quality in the country (Hussain and Mazid 2002; Simonsen et al. 2005).

In an attempt to restoring quality of the hatchery produced seed, the Department of Fisheries (DoF), Government of Bangladesh initiated a Brood Bank Establishment project for the three Indian major carps species in 2008. The objective of the project is to ensure supply of good quality juvenile brood to the hatcheries to develop broodstock. The founder stocks of the broodbank were developed by collecting spawn of *Labeo rohita*, *Catla catla* and *Cirrhinus cirrhosus* from three major river sources- the Halda, the Jamuna, and the Padma. These three rivers have been identified as the spawning ground of the Indian major carps. The collected spawn were randomly distributed among 20 Government owned fish seed multiplication farms across the country and maintained following standard nursing and rearing protocols. The fishes, upon grown up, have been introduced in the induced breeding program of the Government hatcheries for the last few years for multiplication and dissemination.

Genetic variation is important in maintaining the evolutionary potential and fitness of a population (Vandewoestijne et al. 2008). The founder stocks of the hatcheries are finite and are prone to inbreeding and genetic drift- two potential features that can destroy the genetic variability of the hatchery populations over time. Thus, maintenance of genetic variation is the most important concern for the hatchery stock management. The level of success in the hatchery management and also the status of the hatchery brood stock can be assessed by comparing the genetic variability of the hatchery stock with that of a reference natural stock or preferably of the base population (Hansen et al. 2000). Therefore, in order to allow monitoring of the genetic quality of the hatchery stocks in future, it is recommended that the genetic structure of the base population (e.g. Brood bank population) be analyzed with an appropriate molecular marker and the information is archived. Microsatellites, defined as loci of short DNA (2–6 bp) sequences arranged in tandem repeats (simple sequence repeats, SSR) and distributed randomly throughout the genome (Tautz 1989), have been recognized as highly polymorphic markers suitable for genetic characterization and applicable in many studies of ecology, evolution and conservation (O’Connell and Wright 1997; Hansen and Jensen 2005; Abdul-Muneer 2014). Molecular marker-based genotyping can also be used for assessing family relationships and genetic relatedness among individuals of a population. These information have practical implications in behavioral ecology as well as population and conservation genetics (Blouin 2003); and are particularly important for breeding programs (Hansen and Jensen 2005). Several sets of microsatellite DNA markers have been isolated from *L. rohita* (Das et al. 2005; Sahoo et al. 2014) and used for genetic analysis (Alam et al. 2009). The objective of the study was to reveal the population genetic structure and relationship among *L. rohita* of the Jamuna, Padma and the Halda rivers maintained under the Brood Bank of the Department of Fisheries.

## Materials

### Sample collections and extraction of genomic DNA

We collected *L. rohita* brood fish of approximately 2-years-old in July 2010 from three broodstocks maintained under the Brood Bank of the Department of Fisheries, Government of Bangladesh. The broodstocks were developed from spawn of the three major rivers- the Jamuna, the Padma, and the Halda. The Jamuna stock was maintained in Shambhugonj Farm (Mymensingh) (n = 500), the Padma stock was maintained in Rajshahi Farm (n = 500) and the Halda stock was maintained Jangalia Farm (Comilla) (n = 400). A small clip from the caudal fin of 30 randomly selected fish of each of the three river sources was collected and immediately preserved in 95 % ethanol until used for isolation of genomic DNA.

We extracted genomic DNA using the standard proteinase-K digestion, phenol: chloroform: isoamyl alcohol extraction and alcohol precipitation method as described in Islam and Alam (2004). DNA quantity was measured using a spectrophotometer (BioPhotometer plus, Eppendorf, Germany).

### Microsatellite genotyping

For genetic diversity analysis, we used six pairs of microsatellite primers (*Lr1, Lr3, Lr6, Lr12, Lr14b* and *Lr21*) developed from *L. rohita* by Das et al. (2005). The primers were selected based on the number of alleles and polymorphic information content. PCR reaction contained 50 ng template DNA, 0.25 μM of each primer, 0.25 mM of each of the dNTPs, 1.5 μl 10 × reaction buffer containing 1.5 mM MgCl_2_ and one unit of *Taq* DNA polymerase in a total volume of 15 µl. PCR amplifications were performed in a gradient thermal cycler (Master Cycler Gradient, Eppendorf, Germany) with the following temperature profile: 3 min initial denaturation at 94 °C followed by 35 cycles, each of 30 s at 94 °C, 30 s at respective annealing temperature and 1 min at 72 °C. Finally, an additional one cycle of 7 min at 72 °C was added to allow complete elongation of the amplified products. For genotyping, the PCR products were separated on 6 % denaturing polyacrylamide gel containing 19:1 acrylamide:bis-acrylamide and 6 M urea using a Sequi Gen GT sequencing electrophoresis system (BIO-RAD Laboratories, Hercules, CA, USA) and visualized by staining with silver nitrate following the Promega (Madison,WI) protocol. The stained gel plates were scanned and saved in GPEG format and the bands representing particular alleles at the microsatellite loci were scored based on the sizes estimated by the AlphaEase FC, 1D gel image analysis software.

### Statistical analysis of microsatellite data

The genotype data comprising the six microsatellite loci of 90 fish from three populations were subjected to test for null alleles and large allele dropouts or stutter-bands using the software MicroChecker (van Oosterhout et al. 2004). We estimated the polymorphic information content (PIC) for each locus in the complete set of samples using the software CERVUS version 3.0.3 (Kalinowski et al. 2007). As measures of genetic variability in the three populations at six loci, we estimated the population-wise allelic richness (*Ar*), observed heterozygosity (*Ho*) and expected heterozygosity (*He*) (Nei 1987) at each locus by the software FSTAT 2.9.3.2 (Goudet 2001). The exact *p* value for deviation from Hardy–Weinberg expectation at each locus was estimated by the Markov chain method (Guo and Thompson 1992) implemented in the software GENEPOP 4.0 (Rousset 2008) with the following parameters: Dememorization-10000, batches- 1000 and iterations per batch- 10000. The software FSTAT (Goudet 2001) was used for calculating the pair-wise *F*_*ST*_ (Weir and Cockerham 1984) values by means of 10,000 permutations of genotypes. The 95 % confidence interval for each pair-wise *F*_*ST*_ was calculated by bootstrapping on loci with 1000 replications. For all multiple comparisons, the global significance level (0.05) was subjected to sequential Bonferroni corrections (Rice 1989).

For inferring the number of sub-populations, the software STRUCTUE 2.3.2 (Pritchard et al. 2000; Falush et al. 2003) was used with a burn in length of 50,000 and MCMC (Monte-Carlo Markov Chain) iterations of 500,000. Five independent runs were performed for each k value, ranging from 1 to 5. The website based program STRUCTURE HARVESTER (Earl and vonHoldt 2012) was used for determining the number of clusters according to Evanno et al. (2005). We examined the evidence for genetic bottlenecks in each population using the software BOTTLENECK version 1.2.02 (Cornuet and Luikart 1996) under the infinite allele model (IAM), two-phase model (TPM) and stepwise-mutation model (SMM) with 1000 replications.

We reconstructed half- and full-sib families without information on parental genotypes as per maximum likelihood method implemented in the software COLONY 2 (Jones and Wang 2010). A tentative 2.5 % error rate was assumed for all loci, both for allelic dropouts and erroneous sizing of alleles. We compared the family configuration of each sample with that of a sample of the same number (n = 30) of unrelated individuals simulated from the empirical allele frequency data by using the HYBRIDLAB v 1.0 program (Nielsen et al. 2006). Hansen and Jensen (2005) inference was followed for significance in family reconstruction: the sibship reconstruction of a sample was considered ‘significant’ if the number of half- and full-sib families was lower in the real samples compared to the samples of simulated unrelated individuals and if there were more individuals in one or more of the full-sib families generated from real individuals than were observed in any of the full-sib ‘families’ generated from the simulated unrelated individuals.

## Results

### Genetic diversity within population and deviations from Hardy–Weinberg expectation

A total of 28 alleles were detected at six microsatellite loci in the three rohu populations. Examination of genotyping errors using MicroChecker revealed no evidence for null alleles and large allele dropout or stutter-band scoring at any of the six loci. The number of alleles ranged from three (*Lr14b*) to six (*Lr1* and *Lr21*) with an average of 4.66 alleles per locus (Table 1). All six microsatellite loci analyzed in the samples of three river populations were found to be polymorphic (*P*_*95*_) and the polymorphic information contents ranged from 0.646 to 0.808.

**Table 1:** Genetic variation in three populations of *L. rohita*

Locus	Allele sizes (bp)	PIC	Parameters	Population		
				Jamuna	Padma	Halda
*Lr1*	140–164	0.776	*Ar*	6.000	5.000	6.000
			*Ho*	0.833	0.600	0.733
			*He*	0.808	0.762	0.685
			*F*_*IS*_	−0.014	0.229	−0.007
			HWEP	0.0001*	0.0000*	0.0001*
*Lr3*	142–166	0.754	*Ar*	5.000	5.000	5.000
			*Ho*	0.500	0.633	0.767
			*He*	0.762	0.742	0.733
			*F*_*IS*_	0.359	0.163	−0.029
			HWEP	0.0000*	0.0000*	0.0000*
*Lr6*	138–154	0.747	*Ar*	4.000	4.000	4.000
			*Ho*	0.600	0.533	0.567
			*He*	0.736	0.740	0.741
			*F*_*IS*_	0.201	0.295	0.285
			HWEP	0.0033*	0.0045*	0.0035*
*Lr12*	148–178	0.705	*Ar*	4.000	4.000	4.000
			*Ho*	0.700	0.833	0.800
			*He*	0.662	0.727	0.691
			*F*_*IS*_	−0.041	−0.130	−0.142
			HWEP	0.3507NS	0.2827NS	0.0069*
*Lr14b*	184–192	0.646	*Ar*	3.000	3.000	3.000
			*Ho*	0.567	0.667	0.667
			*He*	0.643	0.620	0.598
			*F*_*IS*_	0.135	−0.058	−0.098
			HWEP	0.2589NS	0.0521NS	0.1822NS
*Lr21*	132–168	0.808	*Ar*	6.000	6.000	6.000
			*Ho*	0.767	0.667	0.700
			*He*	0.738	0.761	0.764
			*F*_*IS*_	−0.022	0.140	0.100
			HWEP	0.0010*	0.0261NS	0.0353NS
Mean(±SD) *Ar* across loci				4.667 ± 1.211	4.50 ± 1.048	4.667 ± 1.211
Mean(±SD) *Ho* across loci				0.661 ± 0.127	0.655 ± 0.10	0.705 ± 0.082
Mean(±SD) *He* across loci				0.724 ± 0.061	0.725 ± 0.053	0.702 ± 0.059
Mean(±SD) *F*_*IS*_ across loci				0.103 ± 0.158	0.106 ± 0.165	0.018 ± 0.154

*PIC* Polymorphic information content, *Ar* Allelic richness, *Ho* observed heterozygosity, *He* expected heterozygosity, *F*_*IS*_ inbreeding coefficient, *HWEP* probabilities for Hardy-Weinberg expectation, *NS* not significant

* Significant after sequential Bonferroni corrections (initial k = 3, Rice, 1989)

The observed heterozygosity (*Ho*) ranged from 0.500 to 0.833 and the expected heterozygosity (*He*) ranged from 0.598 to 0.808 (Table 1). The average observed heterozygosity in the Halda population (Ho = 0.705) and the average expected heterozygosity of the Padma population (He = 0.725) were slightly higher than those of the other two populations. The *F*_*IS*_ values ranged from −0.098 to 0.359; the mean *F*_*IS*_ values for the Jamuna, Padma and the Halda populations were 0.103 ± 0.158, 0.106 ± 0.165 and 0.018 ± 0.154 respectively. Significant deviations from Hardy–Weinberg Expectation (HWE) were detected in 11 of the 18 tests (Table 1). The Jamuna and the Halda population deviated at four loci each and the Padma population deviated at three loci.

### Population structure, population differentiation and genetic distance

**Fig. 1:**
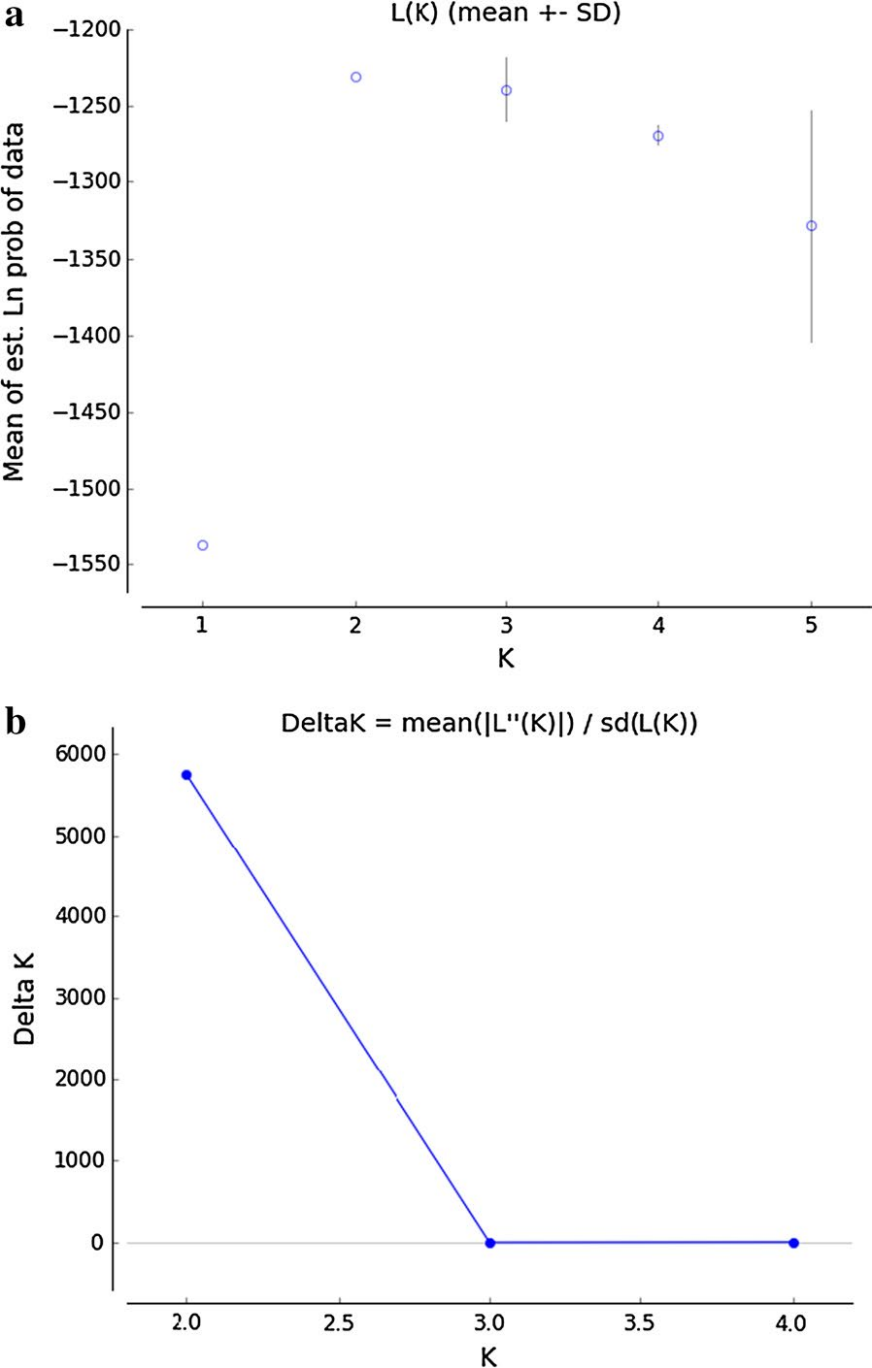
**a** Log probability of data L(K) for each k values ranging from 1 to 5 for the admixture and correlated frequencies model (averaged over five independent runs) for the brood bank collections of *L. rohita* (k = number of cluster). **b** Delta k values for each of the k inferred clusters of *L. rohita* with a maximum value obtained at k = 2

We obtained consistent results across the five independent runs for each k value (k = 1–5). The mean estimated log-likelihood value was found to be the highest for k = 2 (Fig. 1a). Delta k, the quantity of the second order rate of change of the likelihood function estimated by the method of Evanno et al. (2005) was also highest for k = 2 (Fig. 1b). The pair-wise population differentiation (*F*_*ST*_) value between the Padma and the Halda population was the highest (0.0278) and that between the Halda and the Jamuna population was the lowest (0.0057) (Table 2). The *F*_*ST*_ values between the Halda-Padma and Jamuna-Padma population pairs were found to be significant (P < 0.01667). The Nei’s (1972) genetic distance between the Jamuna and the Padma populations was the highest (0.128) while that between the Jamuna and the Halda population was the lowest (0.062) (Table 2).

**Fig. 2:**
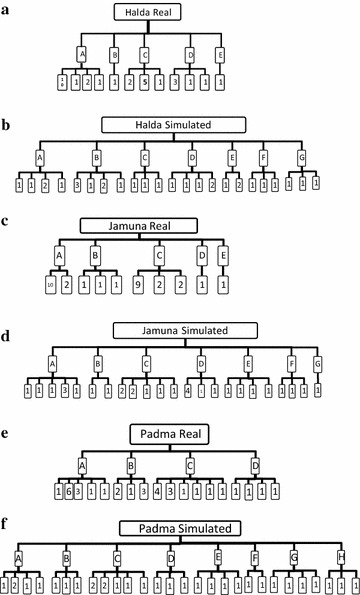
Results of sibship reconstruction grouping the individuals into full-sib families under the corresponding half-sib families (cluster). The *upper hierarchy* of the *figures* denotes the source of the sample, the *second hierarchy* denotes the half-sib families (cluster) and the *third hierarchy* denotes individual full-sib families nested within half-sib families. The number of individuals included to each full-sib family is mentioned. **a** Sibship reconstruction based on real Halda river (HR) individuals, **b** simulated unrelated HR individuals, **c** real Jamuna river (JR) individuals, **d** simulated unrelated JR individuals, **e** real Padma river (PR) individuals, **f** simulated unrelated PR individuals

**Table 2:** Above diagonal: pairwise estimates of *F*_*ST*_ and their associated 95 % confidence interval (±) calculated based on bootstrapping over loci (1000 replications) (P critical = 0.01667, after Bonferroni correction)

Population	Jamuna	Padma	Halda
Jamuna		0.0255* ± 0.0015	0.0057 ± 0.0007
Padma	0.128		0.0278* ± 0.0015
Halda	0.062	0.124	

Below diagonal: Nei’s (1972) genetic distance between population pairs of *L. rohita*

* Significant after sequential Bonferroni corrections (initial k = 3)

### Genetic bottlenecks

For detecting genetic bottlenecks, we applied sign test, Wilcoxon rank test and mode shift tests under IAM, TPM and SMM. Sign test could not detect bottlenecks in any of the three populations (P > 0.0167) while the Wilcoxon sign rank test and the mode-shift test detected recent bottlenecks in all populations under the three mutation models (P < 0.01667) (Table 3).

**Table 3:** Results of bottleneck test for three river stocks of *L. rohita* Broodbank

Population	Sign test			Wilcoxon rank test			Mode shift
	IAM	TPM	SMM	IAM	TPM	SMM	
Jamuna	0.037	0.041	0.043	0.015*	0.015*	0.015*	Shifted
Padma	0.033	0.041	0.043	0.015*	0.015*	0.015*	Shifted
Halda	0.033	0.038	0.044	0.015*	0.015*	0.015*	Shifted

The values indicate probability under the hypotheses of mutation drift equilibruim

* Evidence for recent bottlenecks (P < 0.0167) (after Bonferroni correction)

Shifted mode of allele frequency indicates occurrence of recent genetic bottlenecks

### Family reconstruction

We reconstructed full-sib and half-sib families using Wang’s (2004) method which showed that the number of half-sib families ranged from four (Padma) to five (Halda and Jamuna) and the number of full-sib families ranged from 10 (Jamuna) to 18 (Padma) (Fig. 2a, c, e). We also reconstructed full- and half-sib families from the same number (#30) of unrelated individuals simulated from the gene frequency of the original samples to test the significance of family reconstruction according to Hansen and Jensen (2005). The family reconstructions were found to be significant as the numbers of both the half-sib and full-sib families in the simulated samples were higher in all three populations and several of the putative full-sib families of the real samples included far more individuals than were observed in the simulated data (Fig. 2b, d, f). For example, two full-sib families of the Halda and Jamuna and four full-sib families of the Padma samples contained higher number of individuals than the highest value obtained for the corresponding simulated data of the respective population (Fig. 2).

## Discussion

Establishing a brood bank for aquaculture species may serve two purposes: preserving the genetic variation of the population by maintaining a large random mating population and supporting genetic improvement programs for important production traits. Maintaining genetic variation is very important because the potentials for genetic improvement and fitness of a population are dependent on the existence of genetic variation. Genetic characterization of the founder population would allow monitoring of the brood bank stocks in future to assess the impact of captive breeding on the genetic structure.

In the present study, we have characterized six microsatellite loci in *L. rohita* of the Brood bank stocks collected from three major rivers- the Halda, the Jamuna, and the Padma. The six markers were found to be highly informative as the PICs were >0.500 (Botstein et al. 1980) supporting the suitability for analysis of genetic diversity. Using DNA markers, the population genetic structures of these three river populations had also been studied in the past (Islam and Alam 2004; Alam et al. 2009). Islam and Alam (2004) applying six decamer random primers analyzed a total of 140 *L. rohita* collected from the rivers Jamuna, Padma and Halda and one hatchery while Alam et al. (2009) analyzed 105 fish collected from the same three rivers using four microsatellite markers. However, samples used in all the previous studies were collected randomly and have not been maintained for propagation as a recognized breeding unit.

The objective of establishing the brood bank is to overcome genetic problems and ensure supply of quality brood and fingerlings to be used for aquaculture in the country. If records are maintained about these stocks, the present data could be used to evaluate the changes in genetic parameters of the brood bank stocks of *L. rohita* in future. For evaluating intra-population genetic variation, we analyzed allelic richness, observed heterozygosity, expected heterozygosity and inbreeding coefficient. For inter-population genetic diversity, we analyzed *F*_*ST*_ and genetic distance among population pairs. The observed heterozygosity and expected heterozygosity obtained in the present study were similar to those reported by Sahoo et al. (2014) who analyzing 11 loci in 192 individuals from three rivers and one hatchery stocks reported observed heterozygosity ranging from 0.500 to 0.870 and expected heterozygosity ranging from 0.389 to 878. However, the allelic richness obtained in *L. rohita* populations in the present study were greater than those reported by Das et al. (2005) who analyzed 12 microsatellite loci (including the six used in the present study) in 18 unrelated fish and Alam et al. (2009) who analyzed four microsatellite loci in 105 fish. Sahoo et al. (2014) observed null alleles at locus Lr-12 however we have not detected null alleles at any of the six loci. In fact, null allele is the result of mutation at the priming site which is completely a random process. Therefore, null alleles may be observed in one population but may not in the others even within the same region.

The present study detected significant deviations from the HWE in 61.11 % cases. Alam et al. (2009) reported significant deviation in HWE in 41.66 % in *L. rohita* samples of the same three rivers. The observed heterozygosity (*Ho*) values were higher than the respective heterozygosity (*He*) values expected from observed number of alleles at 50.00 % of the loci in the populations indicating the probability of recent severe reduction in effective population size, a phenomenon popularly termed as genetic bottleneck. The tests for genetic bottlenecks under the assumption of mutation drift equilibrium though not conclusive revealed signs of decline of the individuals in all the populations of *L. rohita*. Bottlenecks were also detected in populations of the other three major carps species *Catla catla* (Basak et al. 2014), *Cirrhinus cirrhosus* (Hasanat et al. 2014) and *Labeo calbasu* (Saha et al. 2010).

The production of major carps in the Padma and the Jamuna river systems in the year 2001–2002 were 187 MT and 157 MT (FRSS 2003) which reduced to only 103 MT and 43 MT respectively in the year 2010–2011 (FRSS 2012). Production of carp spawn in the Halda river also reduced substantially from 2470 kg in 1945 to 234 kg in 2011 with an all-time low of only 20 kg in 2004 (Azadi and Arshad-ul-Alam 2012). This information testify the reduction in population sizes of the three major river systems recognized as natural breeding grounds of the Indian major carps. Population declines in the rivers may be attributed to many reasons such as overexploitation through destructive fishing (fixed gear across the rivers and use of monofilament gill net), habitat destruction, water pollution and siltation. For the Halda river, it should be taken into consideration whether the recruitment in the river is enough as the spawns are collected intensively every year. A stock assessment program (Rahman 2008) supplemented with thorough genetic characterization involving samples from different location and large number of loci would help evaluate the trend of fish population in the rivers, particularly of the Halda river. It is important because, this bottleneck may result in reduction in genetic variation in near future which will ultimately lead to reduction in adaptability to the fluctuating environmental conditions under the climate change scenario (Vandewoestijne et al. 2008). Taken together the signs of bottleneck revealed by the genetic analysis and the trend of production in the major rivers, management measures should be adopted such as temporary ban on fishing particularly in the breeding season to enhance the fisheries.

The *F*_*ST*_ values between different population pairs ranged from 0.0057 to 0.0278, which indicate that the genetic differentiation among the studied populations is low (Balloux and Lugon-Moulin 2002). Hansen et al. (2006) analyzing seven microsatellite loci also reported weaker differentiation between the Jamuna and the Halda river populations compared to the hatchery populations of catla suggesting strong genetic drift in the hatchery stocks.

The number of individuals genotyped and the number of markers used for genotyping play a role in accurate determination of the number of groups. Evanno et al. (2005) opined that calculating delta k using as low as five microsatellite markers, can allow detection of the real number of groups. Regarding sample sizes for microsatellite-based population genetic studies, Hale et al. (2012) proved that a sample size of 25–30 individuals per population was enough for accurately estimating the allele frequencies and expected heterozygosity in a population. Thus, the number of individuals we genotyped from each population (n = 30) and the number of microsatellite markers we used (six nos.) comply with both the conditions for population genetic analysis.

Inbreeding and genetic drift are two major consequences that may deteriorate the genetic quality of a captive population maintained in a hatchery or a brood bank. No inbreeding was detected in the brood bank stocks of *L. rohita* of the three major rivers. However, it may happen when the broods of next generation will be selected. And at the same time care should be taken to avoid any change in gene frequencies due to sampling error, a phenomenon termed as genetic drift. The next generation’s broods should be selected in such a way that the alleles with the minimum frequencies in the base population are not lost because once lost, it may be a loss for ever. The lowest frequency of alleles we detected was 0.017. Since, we are discussing about a brood bank collections, a 99 % guarantee should be ensured to save the rare alleles. And if we want to maintain that guarantee for ten generations, the effective number of breeders (*Ne*) should be at least 344 and for five generations, the Ne should be at least 309 (Tave 1993).

According to Hansen and Jensen (2005), the family reconstruction is considered to be significant if the numbers of families reconstructed from the simulated genotypes were higher than those constructed from original genotype data. Reconstruction of families involving simulated unrelated individuals, based on the allele frequencies of the respective samples, confirmed that much higher numbers of families that include fewer individuals would be expected as compared to the real samples. For instance, the highest number of individuals within a full-sib family in the simulated Halda river (HR) sample was two (Fig. 2b), whereas two full-sib families in the real Halda sample consisted of five and ten individuals (Fig. 2a). Similar trend was also observed in the Jamuna and Padma samples. The low numbers of families found in the real hatchery samples were not due to random grouping of individuals, but should be considered significant (Hansen and Jensen 2005). In a similar study, Hansen et al. (2006) reported fewer numbers of half- and full-sib families in hatchery samples compared to the river counterparts in *Catla catla*.

In conclusion, we have characterized the population genetic structure of the founder stocks of *L. rohita* broodbank originated from three major river sources. These three rivers are considered the principal sources of the Indian major carps. So, the samples are representative of the species from Bangladesh. We have detected genetic bottlenecks in all three river samples of the broodbank collections meaning that the populations apparently have experienced drastic reduction in the recent past. The numbers of families in the sample of all three river were also low indicating a close relationship among the individuals which is not positive for a founder stock. Therefore, it is recommended to collect new batches of fish from various locations of the respective rivers that may broaden the genetic variability of the base population of the brood bank. As some negative points have been exposed from this study, it is strongly recommended to analyze at least 100 fish from each of the rivers with fifteen microsatellite DNA markers to obtain more precise information on genetic status of the brood bank stocks of *L. rohita*. This genetic data would serve as baseline information of the brood bank collections and enable efficient monitoring of the impact of natural and/or human interferences on the river populations of *L. rohita* in future.

## Abbreviations

Ar: allelic richness
C: celsius
DNA: deoxy ribonucleic acids
dNTPs: deoxy ribonucleotide triphosphates
DoF: Department of Fisheries
FAO: Food and Agriculture Organization
FRSS: Fishery Resources Survey System
He: heterozygosity expected
Ho: heterozygosity observed
HWE: Hardy–Weinberg expectation
IAM: infinite allele model
MgCl_2_: magnesium chloride
mM: milli-molar
µM: micro-molar
µl: microliter
MCMC: Monte Carlo Markov Chain
M: molar
PCR: polymerase chain reaction
PIC: polymorphic information content
RFLP: restriction fragment length polymorphism
RAPD: random amplified polymorphic dna
SMM: stepwise mutation model
TPM: two-phase model
